# Can Plant-Based Cheese Substitutes Nutritionally and Sensorially Replace Cheese in Our Diet?

**DOI:** 10.3390/foods14050771

**Published:** 2025-02-24

**Authors:** Andreja Čanžek Majhenič, Alenka Levart, Janez Salobir, Tina Prevc, Tanja Pajk Žontar

**Affiliations:** 1 University of Ljubljana, Biotechnical Faculty, Department of Animal Science, Chair of Dairy Science, Groblje 3, SI-1230 Domžale, Slovenia; andreja.canzek@bf.uni-lj.si; 2 University of Ljubljana, Biotechnical Faculty, Department of Animal Science, Chair of Nutrition, Groblje 3, SI-1230 Domžale, Slovenia; alenka.levart@bf.uni-lj.si (A.L.); janez.salobir@bf.uni-lj.si (J.S.); 3 University of Ljubljana, Biotechnical Faculty, Department of Food Science and Technology, Human Nutrition Group, Jamnikarjeva 101, SI-1000 Ljubljana, Slovenia; tina.prevc@bf.uni-lj.si

**Keywords:** dairy substitute, plant-based cheese, fatty acid composition, sensory quality, nutritional value

## Abstract

Plant-based substitutes for dairy products represent a rapidly developing market worldwide as they become increasingly popular with consumers. This study aimed to determine the nutritional and sensory quality of ten plant-based cheese substitutes labelled ‘classic’/‘original’ purchased on the Slovenian market. The quality was checked using chemical and sensory analysis. When the results of chemical analysis were compared with the nutritional composition of a semi-hard type of cheese, the plant-based cheese substitutes differed greatly. On average, they contained 60 times less protein, 8 times less calcium and 50% more salt per 100 g of product. Considering median values, plant-based substitutes had 200 times less protein, 40 times less calcium, and 58% more salt compared to cheeses. The fatty acid composition was less favourable when compared to a regular semi-hard type of cheese: 50% more saturated fatty acids, almost five times less monounsaturated fatty acids, and only one third of the polyunsaturated fatty acids per 100 g of product, respectively, but no trans fatty acids. Despite some sensory deficiencies (absence of eyes; crumbly, granular, and tough texture; discordant, fatty, and salty taste; foreign odour and pale colour), the sensory quality in this product category was acceptable overall. More research should be conducted in this area to minimise the knowledge gaps in the nutritional composition and sensory quality of plant-based cheese substitutes.

## 1. Introduction

Cheese is an extremely complex food. There are more than 1.000 variants of cheese that can be produced from the milk of different animals using a variety of production and ripening operations [1]. Cheese is generally considered a nutritious food and, as part of a daily, healthy, balanced diet, a rich source of essential nutrients such as proteins, bioactive peptides, amino acids, polyunsaturated fatty acids, conjugated linoleic fatty acid, minerals (calcium, phosphorus, selenium, and zinc) and vitamins (vitamin A, vitamin B2, and B12) [1,2,3].

In recent years, there has been a worldwide trend towards reducing the proportion of food of animal origin and increasing the proportion of food of plant origin in the diet. The reason for this probably lies in people’s increasing concern for animal welfare, environmental protection, and the ultimate need for sustainable food, not only among vegetarians or vegans, but among consumers in general. But the demand for plant-based substitutes for milk and dairy products has a health aspect as well, such as milk protein allergy being more common in (younger) children and the high incidence of lactose intolerance [4]. The last one is said to affect up to 70% [5] or as much as 75% [6] of the world’s population. The proportion of people with lactose intolerance in Europe varies from 4% (Denmark, Ireland) to 56% (Italy) [5]. Approximately 30–40% of people in Slovenia are lactose intolerant [6,7,8]. In North America and Africa, this share is supposed to be higher than 50%, and in some parts of Asia, the share is almost 100% [5]. It is estimated that as many as 15% of European consumers avoid dairy products for health reasons (lactose intolerance, allergy to milk proteins, problems with high cholesterol, and phenylketonuria), lifestyle reasons (vegetarianism and veganism), and concerns about the possible presence of growth hormones or antibiotic residues in milk [9].

People suffering from a cow milk protein allergy and not from lactose intolerance usually do not completely exclude milk and dairy products from their diet but replace cow milk with less allergenic types of milk, such as goat milk [10]. However, for people who suffer from lactose intolerance, lactose-free milk and dairy products are increasingly available. Despite lactose-free alternatives, some people simply choose to eliminate milk and dairy products from their diet completely and switch to a plant-based diet solely. At the same time, these same consumers want to maintain the sensory characteristics they know from foods of animal origin into foods of plant origin [1].

There are more and more products on the market that mimic the sensory properties of foods of animal origin [11]. One of the reasons for the popularisation of plant-based foods certainly is the increase in the number of vegetarians and vegans. In 2022, Germany had the highest proportion of respondents in Europe, aged 18–29 years, who were vegetarians or vegans (13%). Germany was followed by France (10%), Poland (8%), Italy (6%), and Spain (5%). Among the respondents, the proportion of vegetarians was higher than the proportion of vegans in almost all countries, except in France [12]. In Slovenia the last national dietary survey [13] revealed that among adolescents and adults, 1.7% were opportunistic vegetarians (they do not eat red meat but eat fish and/or poultry meat and/or dairy products and/or eggs), 0.4% were vegans, while lacto-ovo vegetarians (they do not eat meat but drink milk and eat dairy products and eggs) accounted for only 0.2% [13].

Plant-based substitutes such as “ice cream”, “yoghurt”, “butter”, and “cheese” account for the largest market share in the category of plant-based substitutes for foods of animal origin and are expected to reach a value of almost USD 4 billion by 2024 [14]. Plant-based cheese accounts for 45% of the plant-based “dairy” group [15]. Many of these plant-based substitutes found on supermarket shelves in the European region of the World Health Organization (WHO) can be classified as ultra-processed foods [11]. However, a review of electronic bibliographic databases (Web of Science, Scopus, and Science Direct) revealed that the technological process of making plant-based cheese substitutes receive insufficient scientific attention.

Despite heavy watering, fertilisation, deforestation, spraying, etc., foods containing little or no animal ingredients are speculated to have a more favourable impact on the environment and climate. The average consumer might, therefore, conclude that such products are inherently less negative, but this is not the case. Studies in the field of the nutritional quality of plant-based foods are limited, and the WHO has called for more research to fill the gaps in our understanding of the relationship between plant-based substitutes, nutritional quality, and health [16].

Our study aimed to evaluate the chemical composition (the content of fatty acids, proteins, fat, sodium, and calcium) and sensory quality of plant-based pre-packaged cheese substitutes, commercially available in Slovenia. Furthermore, as these products resemble most to the full-fat and semi-hard type of cheese, we decided to compare their nutritional composition and sensory quality with Gouda and/or Edam, the most known cheeses from this group. The results will have multiple applications; on one hand, they will contribute to a better understanding of the nutritional and sensory quality of the increasingly popular plant-based cheese substitutes, while, on the other hand, they will encourage producers to improve the ingredients and nutritional value of these products, which could lead to a better quality of plant-based cheese substitutes and thus to the healthier diet for consumers.

## 2. Materials and Methods

### 2.1. Samples

Recently, 11 regular shops, 7 of bigger food retail chains (Mercator, Tuš, Interspar, Müller, E. Leclerc, Hofer, and Lidl) and 4 smaller specialised shops (Kalček, OrCa, Bio shop Norma, and VitaCare) as well as 5 online shops operating in Slovenia, all together 16, formed a putative pool of shops for the purposes of our study. Based on the market analysis overview and to compare plant-based cheese substitutes with the most known semi-hard type of cheeses, we found 10 plant-based cheese substitutes labelled ‘classic’ or ‘original’, in slices. Table 1 shows a list of the samples with their ingredients. 

As each sample was analysed in triplicates, three units of each sample were purchased at three different locations in Slovenia, ensuring independence between samples. Samples purchased from online retailers were ordered from three different addresses. The samples from regular shops were randomly selected from the shelves and purchased as if they were bought by average consumers. The retailers were, therefore, unaware of the selection of the samples and thus had no influence on their procurement and analysis. The collected samples varied in shelf-life span (between 4 weeks and 10 months); therefore, after procurement, they were stored at 4 °C to preserve their original properties as much as possible prior to analyses. All samples were packaged in a modified atmosphere. The sensory analyses were carried out immediately after opening the packages. The temperature of products for sensory evaluation was 14 ± 2 °C. The samples for the chemical analyses were homogenised in a homogeniser (Grindomix GM200, Retsch GmBh and Co., Haan, Germany) using liquid nitrogen to prevent oxidation. The homogenised samples were stored in PE Zip re-closable bags and stored at −80 °C in the dark to prevent deterioration and were analysed within one month. All chemical analyses of individual samples (N = 10) were performed in triplicate (*n* = 30). Data were reported as the mean value ± standard deviation (SD).

### 2.2. Chemical Analysis

Total fat was determined according to the acid hydrolysis method [17]. Prior to acid hydrolysis with 4 mol/L HCl (Soxtec System 2047 Soxcap, Foss, Denmark), the samples were mixed with Celite (Celite 545, Merck, Darmstadt, Germany). After drying, the fat was extracted from hydrolysed samples with petroleum ether in the Soxtec 2050 (Foss, Denmark) extraction system.

The nitrogen content was determined using the Kjeldahl method [18]. To obtain the protein content in plant-based cheese substitutes, the nitrogen content was multiplied by the factor 6.25. Digestion unit K-435, Distillation unit B-324 (both Büchi, Buechi, Flawil, Switzerland), and the Titrino 702 SM (Methrom, Herisau, Switzerland) were used for measurements according to the manufacturer’s instructions.

After ashing the samples by the method of Ash of cheese [19] and dissolution of the inorganic residues in hydrochloric acid, the method of Minerals in animal feed and pet food [20] was used to determine the mineral content either by flame atomic absorption (calcium) or emission spectrometry (sodium). A PerkinElmer Analyst 200 atomic absorption spectrometer (PerkinElmer, Waltham, MA, USA) was used to perform the measurements, according to the manufacturer’s specifications. The salt (NaCl) content was calculated from the results of the analyses for sodium [21].

The fatty acid (FA) composition of the samples was determined by gas chromatographic separation of the FA methyl esters (FAMEs). The FAMEs were prepared according to the procedure developed by Park and Goins [22] without prior extraction of the fats from the sample. An Agilent 6890 GC (Agilent, Santa Clara, CA, USA) equipped with a flame ionisation detector was used for the separation of FAME. Separation of the FAMEs was performed on an Agilent DB-Fatwax UI capillary column (length 30 m, diameter 0.25 mm, film thickness of stationary phase 0.25 μm, Agilent, Santa Clara, CA, USA). An Agilent ChemStation (OpenLab CSD Chemstation edition for GC, version A.02.01[069]) was used for data acquisition and processing. Standard mixtures of FAME standards (GLC 85, GLC 424, GLC 411, and GLC 68A FAME standards, Nu Chek Prep Inc., Elysian, MN, USA) were used to identify and determine the mass fractions of individual FAs in the sample.

### 2.3. Sensory Analysis

**Figure 1:**
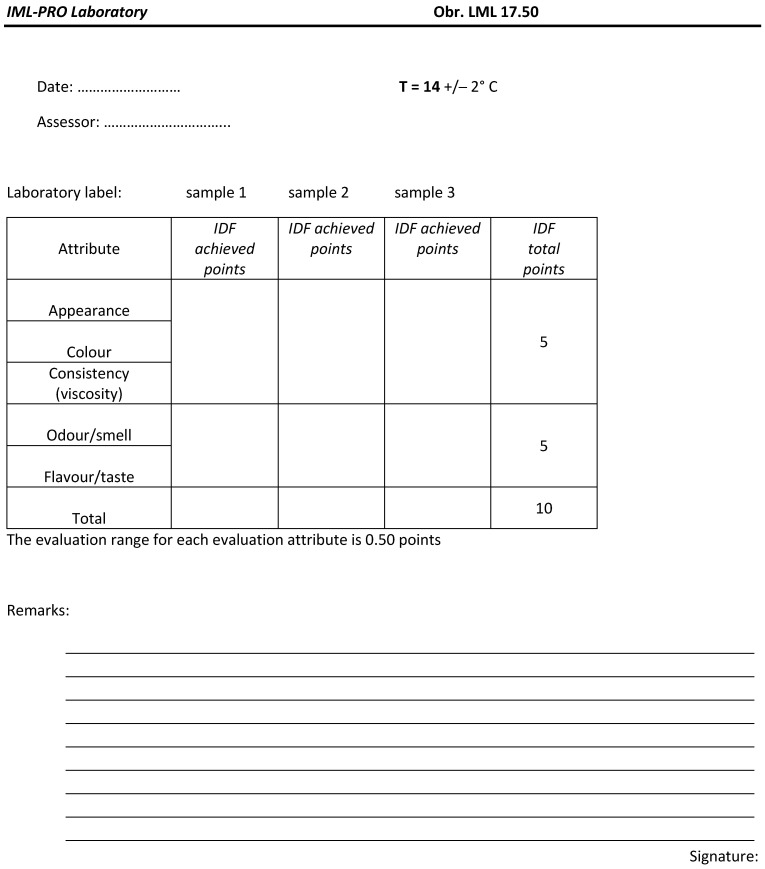
Assessment sheet for sensory evaluation of plant-based cheese substitutes.

The sensory quality of the plant-based cheese substitutes was assessed by a 5-member panel (4 women, 1 man, aged 36–60) from the Biotechnical Faculty (University of Ljubljana, Slovenia) that are all trained and regularly tested. The sensory evaluation of plant-based cheese substitutes was accomplished according to an international standard, published jointly by the International Organization for Standardization (ISO) and the International Dairy Federation (IDF). These standards [23,24] regarding the sensory analysis of milk and milk products, inter alia, cover the following: the sensory quality of cheese is normally assessed according to a 20-point system. But for the purposes of our research, the sensory analysis had to be adapted. Namely, certain characteristics, such as the cross-section, were not considered, because plant-based cheese substitutes do not possess a typical Gouda/Edam cross-sectional appearance, but other characteristics that these products have were considered. Therefore, to evaluate the sensory characteristics of plant-based cheese substitutes, the total amount of achieved points was 10, where 5 points were reserved for general appearance, consistency (texture), and colour, and the other 5 were for smell and taste (Figure 1). As samples are sold in the form of already cut slices, the general appearance comprises the shape and thickness of the slices. The individual sensory characteristics/attributes of the product were assessed with an accuracy of 0.5 points, as agreed by the sensory experts. Each sample was evaluated in triplicate. After each individual sensory assessment, evaluators immediately gave an average score for each product ± standard deviation. This approach of results delivering enabled results regularity due to the specificity of the analysed products (plant-based cheese substitutes). All the detected sensory defects were noted in the evaluation sheets [23] (Figure 1).

A list of the sensory descriptors for smell/odour, aroma, taste, and mouth feel were developed based on literature searches [23] and pre-assessed by the sensory panel to comparatively evaluate the sensory profiles of the samples from different sources.

The final list comprised 4 descriptors for taste (sweet, sour, bitter, and salty), 3 for mouth feel (pungency, astringency, and coating), and 21 for taste and smell/odour and aroma (fruity, grassy, leafy, hay, herbal, woody, walnut, piquant, earthy, metallic, tart, winey, fusty, musty, soapy, rancid, yeasty, proteic, caramel, roast, and burnt). For evaluation of the descriptor intensities, a scale from 0 (undetectable) to 5 (very intense) was applied. Table 2 shows the detailed criteria for sensory evaluation according to ISO standard [24].

The samples were served on ceramic plates at the proper temperature marked with three-digit codes and distributed to the assessors in a balanced presentation order, one at a time. The sensory analysis lasted 1.5 h, and tap water was used to neutralise assessors’ mouth.

### 2.4. Calculation of Nutritional Indices

To evaluate the nutritional value of dietary lipids derived from plants, the following nutritional indices were calculated: the n-6/n-3 FA ratio, the polyunsaturated/saturated FA (PUFA/SFA) ratio, the atherogenic index (AI), and the thrombogenic index (TI) [25].

### 2.5. Statistical Analysis

Descriptive statistics were used to characterise plant-based cheese substitutes. Since most of the data obtained did not follow a normal distribution, comparisons between plant-based cheese substitutes and traditional cheese were conducted using the nonparametric Wilcoxon Mann–Whitney U test. The data analysis was generated using SAS (9.4) software (SAS Institute Inc., Cary, NC, USA). Procedure NPAR1WAY was performed with the “Exact” statement, due to the relatively small sample size.

## 3. Results and Discussion

### 3.1. FA Composition and Amount of Fats

The FA composition and the amount of fat in the plant-based cheese substitutes are presented in Table 3 and Table 4, respectively.

The fat content of the analysed samples ranged from 18.58 g to 24.43 g in 100 g of sample (Table 4).

Only one sample (sample 9) contained less than 20 g of fats in 100 g of sample (Table 4). The FA composition of all samples was very similar (Table 3), which was not surprising as coconut fat was the main ingredient in all samples (written on the declaration). Therefore, most of these products contained predominantly SFAs. The most abundant FA was lauric acid (C12:0, average 46%), followed by myristic acid (C14:0, average 18%), palmitic acid (C16:0, average 9%), caprylic acid (C8:0, average 8%), capric acid (C10:0, average 6%), and stearic acid (C18:0, average 3%). The proportion of mono- and polyunsaturated FAs was low, with the average content of oleic acid (C18:1) being 7% and the proportion of α-linolenic acid (C18:3, n-3) being negligible (0.03%).

### 3.2. Amount of Protein, Salt, and Calcium

The protein, salt, and calcium content of the plant-based cheese substitutes are shown in Table 4. Most of plant-based cheese substitutes contained negligible amounts of protein, on average 0.4 g/100 g per sample. The sample with the highest protein content (sample 3; 1.35 g/100 g) contained pea proteins; in the other samples, the proteins came from sunflower seeds, wholemeal flour, and broad beans, as evident from the declaration.

The salt content in the tested samples varied between 1.44 g and 2.24 g/100 g. Nine out of ten samples contained even more than 1.50 g salt/100 g (Table 4). Salting has a very important technological role in the cheese production, affecting the quality and characteristics of the cheese as follows: it helps in rind formation, reduces moisture in the cheese, and accelerates protein swelling and is thus important for the plasticity of the cheese, has a selective effect on the cheese microbiota, and thus directs the ripening processes; it is also involved in flavour formation and prolongs the shelf life of the cheese. It is obvious that salt is technologically unavoidable in cheese production [26], but there are some reports of unsalted cheese, such as the Italian traditional and local raw milk cheese Pannerone [27]. As salt plays an important technological role in cheese, its quantity cannot be changed at will. It is, therefore, speculated, that the high salt contents in plant-based cheese substitutes probably acts as flavour enhancer, masking other perceptions that are less favourable to consumers.

The majority of samples contained very small amounts of calcium (6.25 to 23.10 Ca mg/100 g) (Table 4). There were two samples containing higher amount of Ca. One sample was declared as enriched with Ca (tricalcium citrate) and contained 604.30 mg Ca/100 g while other one was not but still contained 225.72 mg Ca/100 g. Milk and especially cheese are known sources of calcium, an important mineral that fulfils several important functions in the human body, such as development, growth, and maintenance of bone mass.

### 3.3. Sensory Quality

**Figure 2:**
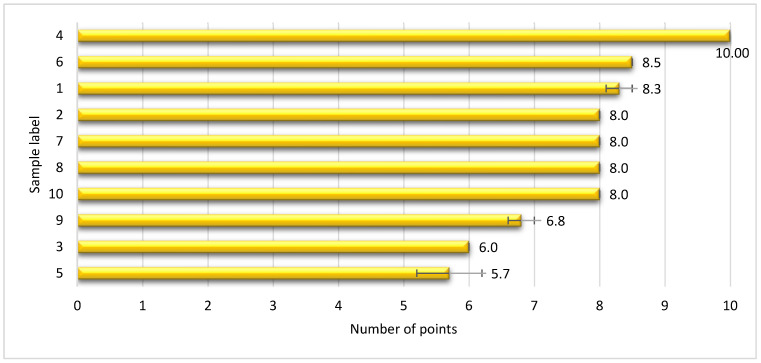
Evaluation of the sensory quality of plant-based cheese substitutes.

The sensory quality of the plant-based cheese substitutes was evaluated using a 10-point system, and their ranking from best to worst is presented in Figure 2.

**Figure 3:**
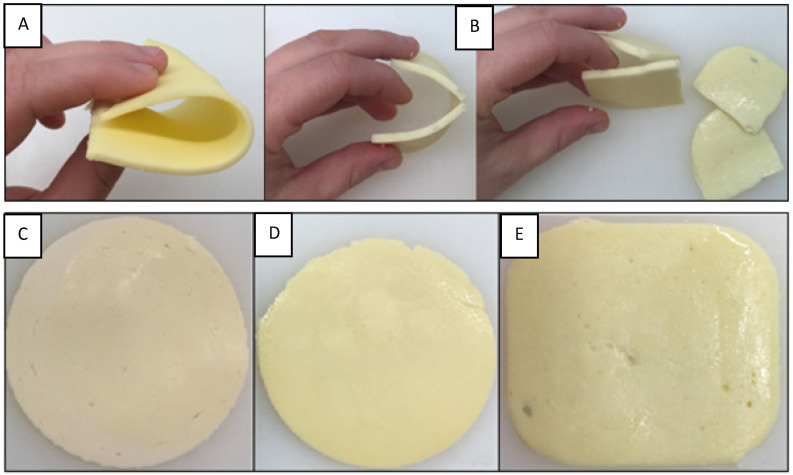
Sensory best sample 4 (**A**)—flexible; sensory worst sample 5 (**B**)—very crumbly. Appearance of plant-based cheese substitutes (samples 9—(**C**), 3—(**D**), and 5—(**E**))—visible air bubbles on the cross-section.

Only sample 4 was scored with the maximum possible score (10). Samples 6 and 1 followed, with an average score of 8.5 and 8.3 points, respectively. They were followed by samples 2, 7, 8, and 10, which had a slightly worse but similar sensory quality and were each rated with 8 points. Samples 9 and 3 followed, with an average rating of 6.8 and 6, respectively. Sample 5 was rated with the lowest average score (5.7 points), which means the worst sensory quality among the analysed plant-based cheese substitutes. Its texture was very crumbly and sandy in the mouth compared to the best rated sample 4 (Figure 3).

Nevertheless, the main difference between plant-based cheese substitutes and semi-hard cheeses is the cross-section, such as the lack of any eyes which are so typical for this group of cheeses and are rarely or densely scattered over the cross-section. The other most common sensory defects found in most of the plant-based cheese substitutes were crumbly/brittle, grainy, and stringy texture, inharmonious, oily/greasy, and salty taste, off-odour, and pale/light colour. The sensory quality of plant-based cheese substitutes was specific and acceptable in the category of these products, but unique compared to the most popular semi-hard cheese variety Gouda/Edam, although consumers expect same, or at least closely similar, sensory characteristics.

### 3.4. Comparison of the Nutritional Value of Plant-Based Cheese Substitutes with Semi-Hard Cheese

Consumers might expect plant-based cheese substitutes to have a similar nutritional value to conventional cheese. Therefore, we compared the median nutritional value of the analysed plant-based cheese substitutes with two most popular cheeses in this group that differ only in fat content in dry matter (DM): ia. three-quarter fat Edam cheese (40% fat in DM), ib. semi-fat Edam cheese (30% fat in DM), and ii. fat Gouda cheese (45% fat in DM) [28]. The 30% fat in DM Edam was chosen because nutritive trends are focused towards consumption of less fatty dairy products.

The nutritional values of the selected semi-hard cheeses are very similar, the expected differences are mainly in the fat content.

The nutrient composition of the plant-based cheese substitutes was statistically significantly different from the cheeses (Table 5). The most important difference was the protein content. There was a significant difference in the amount of protein between the dairy cheese and the plant-based cheese substitute, respectively (*p* < 0.05) (median: 25.6 vs. 0.13 g per 100 g). Milk and dairy products are known sources of protein and as such are an important part of a balanced and everyday human diet [28]. When we compared these data with the results of our study, we concluded that plant-based cheese substitutes proteinaceusly cannot yet replace cheese on an equal basis.

With regard to calcium content, it should be emphasised that, with the exception of two samples with added calcium (Table 4), the plant-based cheese substitutes contained insignificant amounts of calcium. There was a significant difference in the amount of calcium between the dairy cheese and the plant-based cheese substitute, respectively (*p* < 0.05) (median: 0.80 vs. 0.02 g per 100 g) (Table 5). At the same time, the bioavailability of calcium in plant-based cheese substitutes is questionable.

Cheese contains salt, but the amount of salt added during cheese production depends on the variety of cheese. The median amount of salt in semi-hard cheeses such as Gouda or Edam is 1.3 g of salt per 100 g of cheese (Table 5). Indeed, there are significant differences in the salt content of dairy cheeses and plant-based cheese substitutes (*p* < 0.05). The results revealed (Table 4) that all plant-based cheese substitutes from our study exceed this salt content (1.3 g/100 g), and caution should be exercised when consuming plant-based cheese substitutes, as the median of salt content was 0.76 g/100 g higher (58%) than in cheeses (Table 5). Chronic non-communicable diseases, which are also the result of excessive salt intake, cause more than 70% of deaths worldwide and were one of the top ten threats to health in 2019 [29]. To prevent and control these diseases, the WHO had set a global target to reduce dietary salt by 2025 by 30%, which was followed by many countries around the world [30]. For example, the WHO [31] recommends a maximum intake of 5 g of salt per day for the adult population. Slovenia follows this recommendation as well [32]. The median total fat content of the plant-based cheese substitutes was 21.3 g/100 g (average 41% fat in DM). No difference was observed among the amount of total fat between the plant-based cheese substitutes and milk-based cheese options (*p* = 0.6726).

Given that plant-based cheese substitutes contained on average 41% fat in DM, they can be classified as part of the three-quarter-fat group according to the classical cheese classification by percentage of fat in DM [33]. That is why Gouda and Edam cheese with 45% fat in DM, respectively, were chosen to compare the FA composition (Table 6).

The median of FA composition of the plant-based cheese substitutes differed greatly from the cheese (Table 3 and Table 6). The fat in the plant-based cheese substitutes consisted largely of SFA, the proportion of which was as high as 91% (Table 3). The 9% were monounsaturated FAs (MUFAs; 7%) and polyunsaturated FAs (PUFAs; only 2%). Semi-hard cheeses have a similar FA composition, with a more than 1.5 times lower proportion of SFAs compared to the plant-based cheese substitute, while the proportion of MUFAs and PUFAs was higher (the proportion of MUFA was almost 5 times higher and the proportion of PUFA was 3 times higher compared to plant-based cheese substitutes) (Table 3). Both, plant-based cheese substitutes and semi-hard cheeses are dominated by SFAs. However, closer look at the composition revealed that there were significant differences in the amount of individual SFAs between the plant-based cheese substitutes and the selected semi-hard cheeses (*p* < 0.05) (Table 6).

Almost half of the SFAs in the plant-based cheese substitutes was C12:0 (47%), while its share in the selected semi-hard cheeses was only 2%. The proportion of C14:0 in the plant-based cheese substitutes was again higher (18%) than its proportion in the selected semi-hard cheeses (about 9%). The proportion of C16:0 and C18:0 in the plant-based cheese substitutes was low (only 12% in total), whereas these two fatty acids were the most strongly represented in the selected semi-hard cheeses (the proportion of C16:0 is 28% and of C18:0 is 15%). The individual SFAs have a different influence on the concentration of lipoprotein fractions in the plasma. Lauric, myristic, and palmitic FAs are atherogenic, as they increase the concentration of total and LDL cholesterol and thus also the risk of cardiovascular disease, while stearic FA has no such effect [34].

Edam or Gouda (45% fat in DM, respectively) contains 31% oleic FA, while the plant-based cheese substitutes are rather poor with this FA (7%) (statistically significant difference (*p* < 0.05)). Oleic FA has an anti-atherogenic effect as it increases the concentration of HDL cholesterol and slightly decreases the concentration of LDL cholesterol. There is also convincing evidence that replacing SFAs (C12:0*–*C16:0) with oleic acid lowers total and LDL cholesterol levels [34]. Among the PUFAs, present in only 2% in the plant-based cheese substitutes, the most important representatives were linoleic acid (n-6) and α-linolenic acid (n-3), which are essential for humans. As Table 6 shows, the plant-based cheese substitute was not a rich source of essential FAs. Linoleic and α-linolenic acids are precursor molecules for long-chain PUFAs (LCPUFAs) of the n-6 and n-3 type, respectively. LCPUFAs are important for the structure and normal function of cell membranes, while they act as transcription factors and as precursor molecules of tissue hormones (eicosanoids) and regulate inflammatory processes, platelet adhesion and blood clotting, capillary permeability, smooth muscle fibre contractility, the immune system, and secretory hormones [35]. However, their synthesis in humans is limited and depends on the supply of essential FAs and their intake ratio. In recent decades, the Western diet has changed significantly, which is also reflected in a reduced intake of n-3 fatty acids and an increased intake of n-6 fatty acids. As a result, the estimated ratio between n-6 and n-3 fatty acid is 15*–*20:1, which is in contrast to the targeted ratio of about 5:1 or, according to some authors, even a closer ratio such as 2:1 [36]. The n-6/n-3 ratio of the plant-based cheese substitutes varied substantially (Table 3), the highest being 227, and the lowest was 16. A disturbed ratio (in favour of n-6 FAs) is associated with a higher risk of cardiovascular disease, some forms of cancer, type 2 diabetes, osteoporosis, and immune and inflammatory disorders [35]. Table 6 shows that the ratio between linoleic acid (n-6) and α-linolenic acid (n-3) in selected cheeses is ideal, while it deviates significantly from the target ratio in plant-based cheese substitutes. The ratio between n-6 and n-3 FAs in the latter was, on average, 130:1, which means that the meal should be supplemented with foods that are higher in n-3 PUFAs (various seeds—chia, hemp, flaxseed—various oils—hemp, flaxseed, wheat germ—fish—salmon, tuna, rainbow trout, brown trout—microalgae, foods enriched with n-3 fatty acids, and dietary supplements) or foods that have a more ideal ratio (walnuts and walnut oil) [35].

Although trans FAs were not analysed, we can speculate that the samples of plant-based cheese substitutes do not contain trans FAs because they are foods of plant origin, and partially hydrogenated plant oils were not indicated on the label. Conversely, trans FAs in cheese account for about 5% of all FAs produced by microbial metabolism in the rumen of ruminants [3,37].

The nutritionally less favourable FA composition in samples of plant-based cheese substitutes can be additionally supported by the AI and TI, which were high (Table 3), ranging from 12.9 to 15.4 for AI and from 5.7 to 7.3 for TI, respectively. Lower values are desirable, resulting from higher levels of MUFA and PUFA with antiatherogenic or antithrombogenic effects, but at the same time associated with a nutritionally more favourable FA composition for disease prevention [38].

Despite the trans FA presence in cheese, AI and TI were much lower. For comparison, the semi-hard cheese (Gouda or Edam with 45% fat in DM, respectively) has an AI of 1.7 and 1.6 and TI 1.9 or 1.8, respectively [39]. Considering the high AI and TI in plant samples, such products are nutritionally less suitable because their FA composition makes them more prone to deposit fats on the walls of blood vessels and accelerate the formation of blood clots. The effect is even more pronounced when such products are part of an unbalanced diet that does not contain foods with a more favourable FA composition and foods that are a source of essential micronutrients.

### 3.5. Comparison of the Percentage of the Recommended Intake of Micro- and Macronutrients and FA When Consuming the Recommended Amount of Cheese or Plant-Based Cheese Substitutes

**Figure 4:**
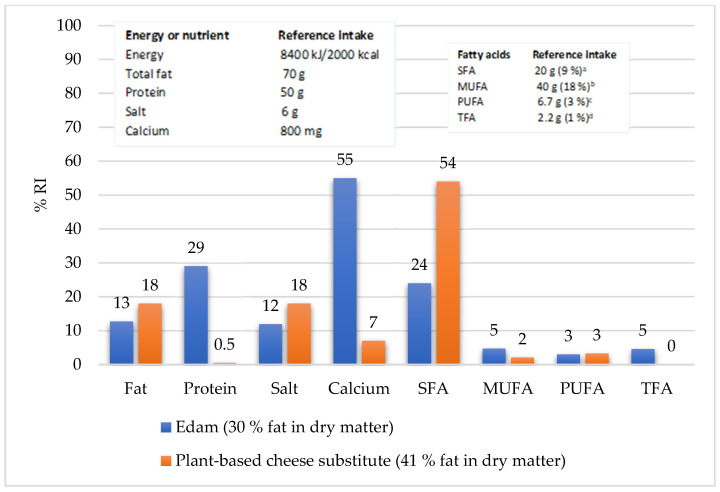
RI percentage for individual nutrients and for each group of fatty acids when consuming 55 g of plant-based cheese substitute and semi-fat Edam cheese with 30% fat in dry matter (its nutritional composition [28]) (recommended daily intakes of energy and selected nutrients for adults from ^a^ Regulation No. 1169/2011, Annex XIII, Part A and Part B [21]; ^b^ calculated − total fats [% Energy] − MUFA [% Energy] − PUFA [% Energy] − TFA [% Energy], ^c^ DGE, 2020, ^d^ FAO, 2010).

It would be interesting to compare to what percentage of the recommended intake (RI) of fat, protein, salt, and calcium would cover an average adult’s needs with consumption of 55 g of semi-hard cheese (Edam with 30% fat in DM—less-fat Edam) or the same amount of plant-based cheese substitute. For this, the 30% fat in DM Edam was chosen because nutritionists recommend consuming reduced-fat dairy products, including cheese. If 55 g of reduced-fat Edam would be replaced with a 55 g of plant-based cheese substitute, the percentage that would cover an average adult’s RI of protein and calcium would be much lower and the percentage of RI of salt and total fat would be greatly exceeded (Figure 4).

With the same intake of a plant-based cheese substitute, an average adult would cover only 0.5% of the RI of protein, 7% for the calcium, and 18% for the total fat and salt. Considering the median values, a plant-based cheese substitute would cover an even lower percentage of the RI of protein and calcium (negligible protein: 0.1% and calcium: 1%). With reduced-fat Edam, an average adult would cover just under a quarter (24%) of the RI of SFA, 5% of the RI of MUFA and TFA, and 3% of the RI of PUFA. Despite the fact that plant-based cheese substitutes do not contain trans FAs, the average adult would still cover slightly more than half (53%) of the RI of SFA, only 2% of the RI of MUFA, and the same percentage of essential FA as with less-fat Edam (Figure 4).

## 4. Conclusions

Chemical composition (the content of FAs, protein, fat, sodium, and calcium) as well as the sensory quality of pre-packaged plant-based cheese substitutes labelled either ‘classic’ or ‘original’ that are commercially available in Slovenia were analysed. The results revealed that most samples had poor nutritional value and average sensory quality.

Contrary to some dietary guidelines which state that cheese can be replaced by plant-based substitutes, our investigation revealed that such substitution could worsen the nutritional profile of our diet due to the insignificant amounts of protein and calcium and increased intake of salt. As coconut fat was the main ingredient in all plant samples, the plant-based cheese substitutes predominantly contain saturated fats, which are associated with increased prevalence for cardiovascular disease risk-factors. To answer the question that we asked the reader in the title, analysed samples of plant-based cheese substitutes that were on the market at the time of our research nutritionally and sensorially cannot replace cheese in our diet. Since the plant ingredients used to make plant-based cheese substitutes differ significantly in composition compared to traditional cheeses, such results are expected.

However, this finding was unexpected as the plant-based cheese substitutes belong to a higher price range and are believed by some consumers to represent “healthier” alternatives to cheese, implying improved nutrient composition in comparison to cheese. This study suggested that more research should be conducted in this field. By using advanced fermentation techniques, microbial cultures, and unique combinations of plant proteins, manufacturers could develop more complex and diverse cheese flavours. In addition, improving the texture to mimic the creaminess and meltability of dairy cheeses is essential for a broader consumer base. Plant-based cheese substitutes can be made with healthier and more sustainable ingredients. Enhancing the nutritional content, e.g., by adding more protein, calcium, and other vitamins and minerals, would offer consumers a more balanced alternative. In addition, reducing additives (e.g., colours, thickeners, humectants, and preservatives) could improve the overall nutritional profile.

## Figures and Tables

**Table 1:** Samples and their ingredients.

Sample	Ingredients
1	Water, refined coconut oil (21%), modified starch (potato, tapioca), calcium citrate, sea salt, olive fruit extract, natural flavouring substances, colours: carotenes, vitamin B12
2	Water, coconut oil (24%), modified starch, sea salt, natural flavouring substances, colours: carotenes, preservatives: sorbic acid, vitamin B12
3	Water, coconut oil *, starch * (corn *, tapioca *), rapeseed oil *, pea proteins *, sea salt, natural flavouring substances (1%), thickeners: locust bean gum, carrageenan, antioxidants: citric acid, turmeric extract *. May contain traces of **gluten, soy, nuts, celery,** and **mustard seeds**.
4	Water, refined coconut oil (21%), modified starch (potato, tapioca), sea salt, olive fruit extract, natural flavouring substances, colours: carotenes
5	Water, coconut oil *, potato starch *, lupine flour *, sea salt, thickeners: xanthan gum *, agar * turmeric extract *, natural flavouring substances (vegan).
6	Water, coconut oil (23%), modified starch, starch, sea salt, natural flavouring substances, olive fruit extract, colours: carotenes, vitamin B12
7	Water, coconut oil (21%), starch, modified starch, sea salt, natural flavouring substances, olive fruit extract, colours: carotenes, vitamin B12
8	Water, starch *, coconut oil (21%) *, sea salt, ground sunflower seeds *, natural flavouring substances, colours: fruit and vegetable concentrate * (carrot, pumpkin, apple)
9	Water, coconut oil * (18%), potato starch *, whole millet flour * (2.8%), thickeners: gellan gum, xanthan gum, natural flavouring substances, rock salt, humectants: plant glycerine *, acids: lactic acid. May contain traces of celery, mustard, soy and lupine. *
10	Water, unrefined coconut oil (20.5%), modified starch, starch, sea salt, natural flavouring substances, preservatives: potassium sorbate, colours: carotenes

* From controlled organic cultivation.

**Table 2:** Numerical discrete interval scale giving the magnitude of deviation in the scoring [24].

Points	Verbal Description
5	No deviation from the pre-established sensory specification
4	Minimal deviation from the pre-established sensory specification
3	Noticeable deviation from the pre-established sensory specification
2	Considerable deviation from the pre-established sensory specification
1	Very considerable deviation from the pre-established sensory specification

**Table 3:** Fatty acid composition (wt %) of plant-based cheese substitutes.

	1	2	3	4	5	6	7	8	9	10	Average	Median	SD	Min	Max
C6:0	0.51 ± 0.03	0.57 ± 0.03	0.62 ± 0.00	0.54 ± 0.01	0.53 ± 0.02	0.55 ± 0.01	0.56 ± 0.04	0.52 ± 0.02	0.53 ± 0.00	0.52 ± 0.01	0.54	0.54	0.03	0.51	0.62
C8:0	7.20 ± 0.17	7.71 ± 0.16	7.67 ± 0.01	7.50 ± 0.14	7.78 ± 0.18	7.49 ± 0.06	7.43 ± 0.23	7.50 ± 0.11	7.16 ± 0.01	7.26 ± 0.02	7.47	7.49	0.21	7.16	7.78
C10:0	5.87 ± 0.04	6.10 ± 0.05	6.13 ± 0.00	5.99 ± 0.08	6.10 ± 0.19	5.99 ± 0.08	5.94 ± 0.07	6.16 ± 0.06	5.78 ± 0.01	5.90 ± 0.00	6.00	5.99	0.13	5.78	6.16
C12:0	46.77 ± 0.04	46.94 ± 0.10	46.20 ± 0.01	46.77 ± 0.13	45.38 ± 0.18	46.81 ± 0.39	46.68 ± 0.10	46.35 ± 0.11	45.66 ± 0.02	46.75 ± 0.02	46.43	46.72	0.53	45.38	46.94
C13:0	0.03 ± 0.00	0.02 ± 0.02	0.03 ± 0.00	0.03 ± 0.00	0.03 ± 0.00	0.03 ± 0.00	0.03 ± 0.00	0.03 ± 0.00	0.03 ± 0.00	0.03 ± 0.00	0.03	0.03	0.00	0.02	0.03
C14:0	18.14 ± 0.07	18.31 ± 0.08	18.03 ± 0.00	17.94 ± 0.02	18.09 ± 0.54	18.48 ± 0.19	18.41 ± 0.28	17.86 ± 0.14	18.61 ± 0.04	18.54 ± 0.01	18.24	18.22	0.27	17.86	18.61
C16:0	9.31 ± 0.04	9.02 ± 0.12	9.34 ± 0.00	9.14 ± 0.09	9.14 ± 0.30	9.33 ± 0.28	9.42 ± 0.08	9.38 ± 0.10	9.82 ± 0.01	9.38 ± 0.00	9.33	9.33	0.22	9.02	9.82
C16:1	0.03 ± 0.00	0.06 ± 0.00	0.03 ± 0.00	0.04 ± 0.00	0.03 ± 0.01	0.03 ± 0.00	0.04 ± 0.00	0.03 ± 0.00	0.03 ± 0.00	0.03 ± 0.00	0.03	0.03	0.01	0.03	0.06
C18:0	2.70 ± 0.01	2.74 ± 0.00	2.72 ± 0.00	2.60 ± 0.02	2.86 ± 0.16	2.66 ± 0.04	2.72 ± 0.02	2.87 ± 0.03	2.89 ± 0.00	3.00 ± 0.00	2.78	2.73	0.12	2.60	3.00
C18:1	7.34 ± 0.04	6.57 ± 0.11	6.90 ± 0.00	7.27 ± 0.20	7.51 ± 0.21	6.73 ± 0.00	6.81 ± 0.43	7.21 ± 0.11	7.07 ± 0.01	6.66 ± 0.01	7.01	6.99	0.32	6.57	7.51
C18:2 n-6	1.85 ± 0.02	1.68 ± 0.00	2.05 ± 0.01	1.92 ± 0.02	2.05 ± 0.17	1.66 ± 0.02	1.71 ± 0.03	1.81 ± 0.03	2.15 ± 0.01	1.69 ± 0.00	1.86	1.83	0.18	1.66	2.15
C18:3 n-3	0.01 ± 0.00	0.03 ± 0.02	0.05 ± 0.00	0.01 ± 0.00	0.13 ± 0.03	0.01 ± 0.00	0.01 ± 0.00	0.02 ± 0.00	0.03 ± 0.00	0.01 ± 0.00	0.03	0.02	0.04	0.01	0.03
C20:0	0.09 ± 0.00	0.09 ± 0.00	0.09 ± 0.00	0.08 ± 0.00	0.10 ± 0.00	0.08 ± 0.00	0.08 ± 0.00	0.09 ± 0.00	0.09 ± 0.00	0.09 ± 0.00	0.09	0.09	0.00	0.08	0.10
C20:1 n-9	0.04 ± 0.00	0.04 ± 0.00	0.04 ± 0.00	0.04 ± 0.00	0.10 ± 0.01	0.05 ± 0.01	0.05 ± 0.01	0.04 ± 0.00	0.04 ± 0.00	0.05 ± 0.00	0.05	0.04	0.02	0.04	0.10
C22:0	0.02 ± 0.00	0.04 ± 0.03	0.02 ± 0.00	0.02 ± 0.00	0.05 ± 0.00	0.02 ± 0.00	0.02 ± 0.00	0.02 ± 0.00	0.02 ± 0.00	0.02 ± 0.00	0.03	0.02	0.01	0.02	0.05
C24:0	0.04 ± 0.00	0.03 ± 0.00	0.04 ± 0.00	0.04 ± 0.00	0.05 ± 0.01	0.03 ± 0.00	0.03 ± 0.00	0.04 ± 0.00	0.04 ± 0.00	0.03 ± 0.00	0.04	0.04	0.01	0.03	0.05
SFA	90.72 ± 0.06	91.62 ± 0.13	90.93 ± 0.01	90.72 ± 0.20	90.16 ± 0.42	91.52 ± 0.02	91.37 ± 0.47	90.88 ± 0.15	90.68 ± 0.01	91.56 ± 0.01	91.02	90.90	0.48	90.16	91.62
MUFA	7.41 ± 0.14	6.67 ± 0.11	6.97 ± 0.00	7.35 ± 0.20	7.65 ± 0.23	6.81 ± 0.00	6.90 ± 0.43	7.29 ± 0.12	7.14 ± 0.02	6.74 ± 0.01	7.09	7.06	0.33	6.67	7.65
PUFA	1.86 ± 0.02	1.72 ± 0.02	2.10 ± 0.01	1.93 ± 0.02	2.18 ± 0.20	1.67 ± 0.02	1.73 ± 0.03	1.83 ± 0.04	2.18 ± 0.01	1.70 ± 0.00	1.89	1.85	0.20	1.67	2.18
n-6 PUFA	1.85 ± 0.02	1.68 ± 0.00	2.05 ± 0.01	1.92 ± 0.02	2.05 ± 0.17	1.66 ± 0.02	1.71 ± 0.03	1.81 ± 0.03	2.15 ± 0.01	1.69 ± 0.00	1.86	1.83	0.18	1.66	2.15
n-3 PUFA	0.01 ± 0.00	0.03 ± 0.02	0.05 ± 0.00	0.01 ± 0.00	0.13 ± 0.03	0.01 ± 0.00	0.01 ± 0.00	0.02 ± 0.01	0.03 ± 0.00	0.01 ± 0.00	0.03	0.02	0.04	0.01	0.13
n-6/n-3	186 ± 16	98 ± 104	39 ± 1	175 ± 40	16 ± 2	227 ± 18	163 ± 12	82 ± 23	81 ± 14	170 ± 3	124	130	70	16	227
AI	13.9	15.4	14.1	13.8	12.9	15.3	15.1	13.9	13.9	15.4	14.4	14.0	0.9	12.9	15.4
TI	6.5	7.0	6.4	6.4	5.7	7.1	7.0	6.5	6.6	7.3	6.7	6.6	0.5	5.7	7.3

SFA: saturated fatty acids; MUFA: monounsaturated fatty acids; PUFA: polyunsaturated fatty acids; AI: atherogenic index; TI: thrombogenic index, n-6/n-3: n-6 PUFA/n-3 PUFA ratio; Results represent mean ± SD.

**Table 4:** Results of chemical analysis of plant-based cheese substitutes.

Sample	Dry Matter (g/100 g) $\overset{-}{x}$ ± SD	Fat Content (g/100 g) $\overset{-}{x}$ ± SD	Protein Content ^a^ (g/100 g) $\overset{-}{x}$ ± SD	Calcium (mg/100 g) $\overset{-}{x}$ ± SD	Sodium (mg/100 g) $\overset{-}{x}$ ± SD	Salt ^b^ (g/100 g) $\overset{-}{x}$ ± SD
1	51.42 ± 0.19	21.32 ± 0.32	0.01 ± 0.01	604.30 ± 4.96	897.34 ± 6.07	2.24 ± 0.02
2	50.38 ± 0.82	24.42 ± 0.65	0.05 ± 0.05	6.25 ± 0.13	725.86 ± 17.23	1.81 ± 0.04
3	43.91 ± 0.30	21.94 ± 0.27	1.35 ± 0.05	225.72 ± 8.21	681.27 ± 13.35	1.70 ± 0.03
4	49.05 ± 0.70	21.28 ± 0.75	0.11 ± 0.03	23.10 ± 1.54	796.56 ± 30.75	1.99 ± 0.08
5	41.26 ± 2.27	20.79 ± 0.54	1.10 ± 0.12	18.78 ± 1.31	615.64 ± 23.51	1.54 ± 0.06
6	49.15 ± 0.64	23.36 ± 0.21	0.08 ± 0.01	14.01 ± 0.78	863.60 ± 49.37	2.16 ± 0.12
7	47.94 ± 0.70	21.24 ± 0.30	0.10 ± 0.02	11.41 ± 0.44	848.23 ± 19.33	2.12 ± 0.05
8	54.03 ± 0.72	20.66 ± 0.39	1.00 ±0.04	20.28 ± 2.03	850.87 ± 25.23	2.13 ± 0.06
9	42.00 ± 0.36	18.58 ± 0.29	0.52 ± 0.01	21.78 ± 0.59	576.81 ± 10.53	1.44 ± 0.03
10	49.02 ± 0.52	23.43 ± 0.14	0.16 ± 0.02	12.92 ± 0.27	873.83 ± 56.89	2.18 ± 0.14
Average	47.81	21.70	0.46	95.85	773.00	1.93
Median	49.03	21.30	0.13	19.53	822.39	2.06
SD	4.15	1.68	0.50	190.5	115.47	0.29
min	41.25	18.58	0.05	6.25	576.81	1.44
max	54.03	24.43	1.35	604.30	897.34	2.24

^a^ When converting nitrogen to protein, we used a factor of 6,25; ^b^ “salt” means the salt content equivalent calculated by the following formula: salt (g) = sodium (mg) × 2.5/1000 [21].

**Table 5:** Comparison of the median nutritional value (g/100 g) of ten plant-based cheese substitutes * with three samples of regular semi-hard type of cheese that differ in fat content in dry matter (g/100 g) [28].

	Plant-Based Cheese Substitute * (N = 10)	Cheese ** (N = 3)	
	Median (Min–Max)	Median (Min–Max)	*p*-Value
Dry matter	49.0 (41.2–54.0)	54.0 (50.9–55.2)	0.0519
Protein	0.13 (0.05–1.35)	25.6 (25.0–25.9)	<0.05
Fat	21.3 (18.6–24.4)	23.4 (16.2–25.4)	0.6726
Salt	2.06 (1.44–2.24)	1.30 (1.30–1.30)	<0.05
Calcium	0.02 (0.01–0.60)	0.80 (0.80–0.80)	<0.05

* Plant-based cheese substitute contained on average 41% of fat in DM; ** Cheese: Gouda (45% fat in DM), Edam (40% fat in DM), and Edam (30% fat in DM); statistical significance is considered when *p*-value < 0.05.

**Table 6:** Comparison of the proportion (mass fraction [%]) of fatty acids and ratio of essential fatty acids in ten plant-based cheese substitutes and two cheeses.

	Plant-Based Cheese Substitutes * (N = 10)	Cheese ** (N = 2)	
	Median (Min–Max)	Median (Min–Max)	*p*-Value
C12:0 (lauric fatty acid)	46.7 (45.4–46.7)	2.14 (2.03–2.24)	<0.05
C14:0 (myristic fatty acid)	18.2 (17.9–18.6)	9.14 (8.87–9.41)	<0.05
C16:0 (palmitic fatty acid)	9.33 (90.2–9.82)	28.2 (27.9–28.4)	<0.05
C18:0 (stearic fatty acid)	2.73 (2.60–3.00)	14.7 (14.5–14.9)	<0.05
C18:1 (oleic fatty acid)	6.99 (6.57–7.51)	30.8 (30.3–31.2)	<0.05
C18:2 n-6 (linoleic fatty acid)	1.83 (1.66–2.15)	2.42 (2.40–2.43)	<0.05
C18:3 n-3 (α-linolenic fatty acid)	0.02 (0.01–0.13)	0.96 (0.95–0.97)	<0.05
SFA (saturated fatty acids)	91.0 (90.2–91.6)	57.1 (56.6–57.5)	<0.05
MUFA (monounsaturated fatty acids)	7.06 (6.67–7.65)	33.9 (33.5–34.3)	<0.05
PUFA (polyunsaturated fatty acids)	1.85 (1.67–2.18)	5.69 (5.64–5.74)	<0.05
n-6 PUFA	1.83 (1.66–2.15)	2.85 (2.82–2.87)	<0.05
n-3 PUFA	0.02 (0.01–0.13)	0.96 (0.95–0.97)	<0.05
n-6/n-3 PUFA ratio	130 (16.1–227)	2.95 (2.88–3.03)	<0.05

* average proportion of fatty acids in all analysed plant-based cheese substitutes (on average 41% of fat in DM); ** average proportion of fatty acids in Edam (45% fat in DM) and Gouda (45% fat in DM) cheese; statistical significance is considered when *p*-value < 0.05.

## Data Availability

The original contributions presented in this study are included in this article; further inquiries can be directed to the corresponding author.

## Abbreviations

The following abbreviations are used in this manuscript:

WHO	World Health Organization
FA	Fatty acid
FAME	Fatty acid methyl esters
ISO	International Organization of Standardization
IDF	International Dairy Federation
AI	Atherogenic index
TI	Thrombogenic index
SFA	Saturated fatty acid
MUFA	Monounsaturated fatty acid
PUFA	Polyunsaturated fatty acid
TFA	Trans fatty acids
LCPUFA	Long-chain polyunsaturated fatty acids
WT %	Weight percentage
DM	Dry matter
HDL	High-density lipoprotein
LDL	Low-density lipoprotein
RI	Recommended intake

## References

[1] Grossmann L., McClements D.J. (2021). The science of plant-based foods: Approaches to create nutritious and sustainable plant-based cheese analogs. Trends Food Sci. Technol..

[2] Manuelian C.L., Currò S., Penasa M., Cassandro M., De Marchi M. (2017). Characterization of major and trace minerals, fatty acid composition, and cholesterol content of protected designation of origin cheeses. J. Dairy Sci..

[3] Jerónimo E., Malcata F.X., Caballero B., Finglas P.M., Toldrá F. (2016). Cheese: Composition and health effects. Encyclopedia of Food and Health.

[4] The Growing Acceptance of Veganism. https://www.forbes.com/sites/janetforgrieve/2018/11/02/picturing-a-kindler-gentler-world-vegan-month/.

[5] Lule V.K., Garg S., Tomar S.K., Caballero B., Finglas P.M., Toldrá F. (2016). Food Intolerance: Lactose intolerance. Encyclopedia of Food and Health.

[6] Silva A.R.A., Silva M.M.N., Ribeiro B.D. (2019). Health issues and technological aspects of plant-based alternative milk. Food Res. Int..

[7] Karas Kuželičko N., Lukač-Bajalo J. (2005). Genetika laktozne intolerance in pogostost polimorfizma-13910C> T v slovenski populaciji. Farm. Vestn..

[8] Šmid E. (2012). Laktozna intoleranca. Slov. Pediatr..

[9] Mäkinen O.E., Wanhalinna V., Zannini E., Arendt E.K. (2016). Foods for special dietary needs: Non-dairy plant-based milk substitutes and fermented dairy-type products. Crit. Rev. Food Sci. Nutr..

[10] Ballabio C., Chessa S., Rignanese D., Gigliotti C., Pagnacco G., Terracciano L., Fiocchi A., Restani P., Caroli A.M. (2011). Goat milk allergenicity as a function of αS1-casein genetic polymorphism. J. Dairy Sci..

[11] Wickramasinghe K., Breda J., Berdzuli N., Rippin H., Farrand C., Halloran A. (2021). The shift to plant-based diets: Are we missing the point?. Glob. Food Secur..

[12] Share of Young Adults Who Are Vegetarian or Vegan in Selected European Countries in 2022. https://www.statista.com/forecasts/768475/vegetarianism-and-veganism-among-young-adults-in-selected-european-countries.

[13] Gregorič M., Blaznik U., Fajdiga Turk V., Delfar N., Korošec A., Lavtar D., Zaletel M., Koroušić Seljak B., Golja P., Zdešar Kotnik K. (2019). Različni Vidiki Prehranjevanja Prebivalcev Slovenije (v Starosti od 3 Mesecev do 74 Let).

[14] Grasso N., Roos Y.H., Crowley S.V., Arendt E.K., O’Mahony J.A. (2021). Composition and physicochemical properties of commercial plant-based block-style products as alternatives to cheese. Future Foods.

[15] Non-Dairy Cheese Market Research Report—Global Forecast Till 2024. https://www.marketresearchfuture.com/reports/non-dairy-cheese-market-3114.

[16] Plant-Based Diets and Their Impact on Health, Sustainability and the Environment: A Review of the Evidence. https://www.who.int/europe/publications/i/item/WHO-EURO-2021-4007-43766-61591.

[17] Horowitz W., AOAC (2000). Method No. 948.15 Fat (crude) in seafood. Acid hydrolysis method. Official Methods of Analysis of AOAC International.

[18] Horowitz W., AOAC (2000). Method No. 920.123 Nitrogen in cheese. Official Methods of Analysis of AOAC International.

[19] Horowitz W., AOAC (2000). Method No. 935.42 Ash of cheese. Official Methods of Analysis of AOAC International.

[20] Horowitz W., AOAC (2000). Method No. 968.08 Minerals in animal feed and pet food. Official Methods of Analysis of AOAC International.

[21] Regulation (EU) No 1169/2011 of the European Parliament and of the Council of 25 October 2011 on the Provision of Food Information to Consumers. https://eur-lex.europa.eu/legal-content/EN/TXT/?uri=CELEX%3A02011R1169-20180101.

[22] Park P.W., Goins R.E. (1994). In situ preparation of fatty acid methyl esters for analysis of fatty acid composition in foods. J. Food Sci..

[23] ISO/IDF (2023). Milk and Milk Products—Sensory Analysis—Part 2: Methods for Sensory Evaluation.

[24] ISO/IDF (2023). Milk and Milk Products—Sensory Analysis—Part 3: Method for Evaluation of Compliance with Product Specifications for Sensory Properties by Scoring.

[25] Ulbricht T.L., Southgate D.A. (1991). Coronary heart disease: Seven dietary factors. Lancet.

[26] Guinee T.P., Fox P.F., McSweeney P.L.H., Fox P.F., Cotter P.D., Everett D.W. (2017). Salt in Cheese: Physical, Chemical and Biological Aspects. Cheese—Chemistry, Physics & Microbiology.

[27] Mucchetti G., Ghiglietti R., Locci F., Francolino S., Bonvini B., Remagni M.C., Zago M., Iezzi R., Carminati D. (2009). Technological, microbiological and chemical characteristics of Pannerone, a traditional Italian raw milk cheese. Dairy Sci. Technol..

[28] Souci S.W., Fachmann W., Kraut H. (2000). Food Composition and Nutrition Tables.

[29] McLean R.M., Petersen K.S., Arcand J., Malta D., Rae S., Thout S.R., Trieu K., Johnson C., Campbell N.R.C. (2019). Science of Salt: A regularly updated systematic review of salt and health outcomes studies (April to October 2018). J. Clin. Hypertens..

[30] Global Action Plan for the Prevention and Control of Noncommunicable Diseases 2013–2020. https://iris.who.int/bitstream/handle/10665/94384/9789241506236_eng.pdf;jsessionid=E117A439EE79A9EBD7467E292C323BA2?sequence=1.

[31] WHO Global Report on Sodium Intake Reduction. https://www.who.int/publications/i/item/9789240069985.

[32] Sol in Zdravje. https://nijz.si/zivljenjski-slog/prehrana/sol-in-zdravje/.

[33] Codex Alimentarius (1973). General Standard for Cheese.

[34] Fats and Fatty Acids in Human Nutrition: Report of an Expert Consultation. https://www.fao.org/fileadmin/user_upload/nutrition/docs/requirements/fatsandfattacidsreport.pdf.

[35] Saunders A.V., Davis B.C., Garg M.L. (2013). Omega-3 polyunsaturated fatty acids and vegetarian diets. Med. J. Aust..

[36] Søborg Husted K., Bouzinova E.V. (2016). The importance of n-6/n-3 fatty acids ratio in the major depressive disorder. Medicina.

[37] Verruck S., Balthazar C.F., Rocha R.S., Silva R., Esmerino E.A., Pimentel T.C., Queiroz Freitas M., Silva M.C., Gomes da Cruz A., Schwinden Prudencio E. (2019). Chapter Three—Dairy foods and positive impact on the consumer’s health. Adv. Food Nutr. Res..

[38] Reis Lima M.J., Fontes L., Bahri H., Veloso A.C.A., Teixeira-Lemos E., Peres A.M. (2020). Fatty acids profile of Serra da Estrela PDO cheeses and respective atherogenic and thrombogenic indices. Nutr. Food Sci..

[39] Koman Rajšp M., Stibilj V. (2000). Fatty acid composition of edam, emmental and gouda cheeses produced in Slovenia in autumn 1997. Acta Agric. Slov..

